# The De Ritis and Neutrophil-to-Lymphocyte Ratios May Aid in the Risk Assessment of Patients with Metastatic Renal Cell Carcinoma

**DOI:** 10.1155/2018/1953571

**Published:** 2018-12-18

**Authors:** Sung Han Kim, Eun Young Park, Jungnam Joo, Jinsoo Chung

**Affiliations:** ^1^Department of Urology, Center for Prostate Cancer, National Cancer Center, Goyang, Republic of Korea; ^2^Biometrics Research Branch, Research Institute and Hospital, National Cancer Center, Goyang, Republic of Korea

## Abstract

**Purpose:**

This study aimed to determine whether baseline blood inflammatory markers can predict progression-free survival (PFS) and overall survival (OS) in patients with metastatic renal cell carcinoma (mRCC).

**Methods:**

The study included 158 patients with mRCC treated with first-line targeted therapy between 2002 and 2016. A multivariable cox proportional hazards model identified inflammatory factors that predict PFS and OS. Using bootstrap method, new prognostic model compared with Heng and modified MSKCC risk model (mMSKCC). The effect of inflammatory factors were investigated by comparing increased C-index adding significant inflammatory factors to Heng and mMSKCC model.

**Results:**

On multivariable analysis, nephrectomy (HR 0.48), NLR (HR 1.04), were significant risk factors for PFS; nephrectomy (HR 0.38), hemoglobin (HR 1.71), alkaline phosphatase (HR 1.73), NLR (HR 1.01) and DRR (HR 1.34), were significant factors for OS (p<0.05). Our new model that incorporated NLR and DRR had higher (though insignificant) predictability (C-index=0.610) than mMSKCC risk model (C-index=0.569) in PFS and significantly better predictability (C-index=0.727) than Heng and mMSKCC risk model (C-index, 0.661, 0.612, respectively) in OS. Adding inflammatory factors to the Heng criteria (C-index, 0.697 for OS) and MSKCC (0.691 for OS) tended to improve their predictive abilities.

**Conclusions:**

The NLR and DRR may increase predictive ability compared to the established Heng and mMSKCC risk models in mRCC.

## 1. Introduction

Patients with metastatic renal cell carcinoma (mRCC) generally show poor prognoses; the 5-year survival rate is 8–20% [1–4]. Clinicians use several prognostic models to stratify patients and determine optimal therapeutic strategies; these include the International Metastatic Renal Cell Carcinoma Database Consortium (IMDC, also known as Heng) Model [5] and Memorial Sloan-Kettering Cancer Center (MSKCC/Motzer score) model [4]. The Heng prognostic model incorporates the Karnofsky performance status, corrected serum calcium, hemoglobin, time from diagnosis to treatment, platelets, and neutrophils [5], whereas the MSKCC model incorporates lactate dehydrogenase, corrected serum calcium, Karnofsky performance status, hemoglobin, and time from initial diagnosis to commencing therapy [6]. However, both models have been cited for their shortcomings and inaccuracies in predicting mRCC prognosis.

Recent scientific improvements have allowed for more thorough examinations of the pathophysiologies of various cancers, including RCC. Such advances have elucidated the importance of the tumor microenvironment, including the host inflammatory immune response and cellular turnover metabolism, in carcinogenesis and tumor progression, especially in RCC [7, 8]. Tumors tend to create microenvironments that promote inflammatory cell proliferation and produce a greater amount of immune response mediators [9]. Laboratory markers of systemic inflammation are among the many prognostic biomarkers identified in RCC, irrespective of the localized or metastatic state of the tumor. C-reactive protein [10], neutrophil-to-lymphocyte ratio (NLR) [11], lymphocyte-to-monocyte ratio [12], and platelet-to-lymphocyte ratio (PLR) [13] have been identified as independent prognostic variables in treatment-naïve patients with RCC [2, 5]. Additionally, recent studies showed that the De Ritis ratio (DRR), which is the ratio of aspartate transaminase (AST) to alanine transaminase (ALT), is indicative of cellular metabolism and cancer cell turnover [14].

The assessment of blood-based markers of inflammatory and metabolic responses in patients with cancer provides a simple and cost-effective evaluation method in clinical practice. Therefore, we investigated the prognostic value of systemic inflammatory markers as well as AST/ALT-related parameters and evaluated those that may be useful in improving survival stratification offered by the current Heng and MSKCC risk models in patients with mRCC treated with targeted therapy.

## 2. Materials and Methods

### 2.1. Ethical Statements

This retrospective study was approved by the Institutional Review Board of the National Cancer Center (No. NCC2015-0087), which waived the requirement for written informed consent. Patient data were anonymized and deidentified prior to analysis. Study procedures were performed in accordance with the guidelines of the Declaration of Helsinki.

### 2.2. Study Design and Patients

Between June 2002 and January 2016, 158 consecutive patients with mRCC treated with first-line vascular endothelial growth factor-targeted therapy (sorafenib, sunitinib, pazopanib, or axitinib) were retrospectively extracted from the prospectively collected kidney cancer database, in which all baseline demographics and clinical and laboratory data, including systemic inflammatory marker information, were prospectively collected. All RCC diagnoses were based on the histological analyses of specimens obtained at nephrectomy, renal biopsies, and/or biopsies acquired from metastatic sites.

### 2.3. Response Assessment

Therapy was administered until disease progression, unacceptable toxicity, or cessation upon the directive of the physician (J.C.). Responses were evaluated using the Response Assessment Criteria in Solid Tumors version 1.1. Progressive disease was defined as a 20% increase in the sum of the products of all measurable lesions, appearance of any new lesions, or reappearance of any lesion that had previously disappeared.

### 2.4. Statistical Analysis

The baseline clinical and inflammatory factors were summarized in Table 1. Progression-free survival (PFS) duration was defined to date of initiation of therapy to date of progression of disease and overall survival (OS) duration was defined to date of initiation of therapy to date of death or last follow up date, respectively. The multivariable Cox proportional hazards model was used to examine the effect of inflammatory factors on prognosis of patients. Each clinical and inflammatory factors with p-value ≤0.15 in univariable analysis were included into multivariable model. Inflammatory factors were used by itself (neutrophil, lymphocyte, ALT, AST) or ratios (NLR, DRR). The final model was proposed using backward selection with an elimination criterion of p-value > 0.05. To compare the predictive ability of new prognostic model with Heng and mMSKCC risk models, 2000 bootstrap samples were used to calculate the C-index of each model. The mean and 95% confidence intervals of difference of C-index were presented. In addition, the C-index of model adding significant inflammatory factors to Heng and mMSKCC risk model was compared to previously that of Heng and mMSKCC risk model. All statistical results were presented as hazard ratio (HR) with 95% confidence intervals. P<0.05 was considered statistically significant. All analyses were performed using R project (version 3.3.3) and SAS version 9.4 (SAS Institute Inc., Cary, NC, USA).

### 2.5. Dichotomization of Inflammatory Variables

We individually examined the impact of baseline markers of systemic inflammation (hemoglobin, platelets, neutrophils, lymphocytes, LDH, corrected Ca, albumin, alkaline phosphatase, AST, ALT) on PFS and OS. These markers were analyzed as categorical variables. Dichotomization of these variables was based on the upper (platelets, neutrophils, LDH, corrected Ca, alkaline phosphatase, AST, ALT) or lower (hemoglobin, albumin and lymphocytes) ranges of normal measurements. No widely accepted cut-off points for NLR, and DRR were previously adopted [15, 16]; therefore, we analyzed these variables as continuous variables.

## 3. Results

### 3.1. Baseline Characteristics

The mean patient age (when commencing treatment) and treatment duration were 58.6 (standard deviation [SD] 10.6) years and median treatment duration was 4.8 months. Metachronous mRCC (61.4%) and male sex (78.5%) were dominant, and 89 of 158 patients (56.3%) had a history of nephrectomy. The baseline proportions of the favorable, intermediate, and poor risk groups according to the MSKCC criteria were 12.2%, 70.8%, and 17%, respectively; those according to the Heng criteria were 10.6%, 71.2%, and 18.2%, respectively. The progression rate was 81.7% after first-line targeted therapy. The patients' baseline data are described in Table 1.

### 3.2. Significant Prognostic Risk Factors for PFS and OS

Univariable analysis showed that metachronous type (hazard ratio [HR] 0.64), nephrectomy (0.48), DFI≤ 1(1.76), Heng (2.00, 2.84), Platelet (1.95), Albumin (1.78), NLR (1.03), AST (2.56) were significantly associated with PFS (p<0.05). More factors were significant in OS univariable analysis, with metachronous type (0.48), nephrectomy (0.34), DFI≤ 1 (2.04), mMSKCC (1.77, 2.83), Heng (3.11, 6.46), Liver mets (1.92), Hb (2.04), Platelet (2.51), Neutrophil (2.17), Lymphocyte (1.71), Albumin (3.77), Alkaline phosphatase (1.83), NLR (1.06), AST (3.60) and DRR (1.39). In multivariable analysis, nephrectomy (HR 0.48) and NLR (HR 1.04) were associated with PFS(p<0.05) (Table 2).

Multivariable models using inflammatory factors as ratio (NLR, DRR) were better predictive ability. Nephrectomy (HR, 0.48) and NLR (1.04) were significant prognostic factors in PFS and nephrectomy (HR 0.38), Hb (1.71), alkaline phosphatase (1.73), NLR (1.07) and DRR (1.34) were also significant factors in OS (p<0.05) (Table 3).

### 3.3. Modeling New Prognostic Risk Criteria for PFS

Two new risk models were created using significant risk factors for PFS, including treatment itself or ratio (Table 4). Model A used inflammatory factors itself (neutrophil, lymphocyte, ALT, AST), and model B used ratio (NLR, DRR). The model consisted of nephrectomy and AST (Model A: C-index 0.594) or nephrectomy and NLR (Model B: C-index 0.610) show no significant differences (mean difference 0.017, 95% CI -0.021 to 0.057) using 2000 bootstrap samples. When comparing the 2 models with the Heng and mMSKCC risk models, 2 models did not show better predictive ability than Heng or mMSKCC risk models. To investigate the effect of inflammatory factors, the models with adding significant inflammatory factor were analyzed. No significant increases in C-index by adding inflammatory factors to established Heng or mMSKCC risk models (p>0.05).

### 3.4. Modeling New Prognostic Risk Criteria for OS

The same methods were applied to derive new OS prediction models. Model A included nephrectomy, liver metastasis, hemoglobin, neutrophil, and alkaline phosphatase, which were significant factors for OS multivariate analysis (Table 3). Model B incorporated nephrectomy, hemoglobin, NLR, alkaline phosphatase, and DRR. Models A and B had Harrell's C-indices of 0.708 and 0.727, respectively, with no significant difference (the mean difference was 0.02, 95% CI -0.011 to 0.058, Table 5). Compared to the Heng (C-index, 0.661) risk model, Model B was significantly better predictive ability (mean difference was -0.055, 95% CI -0.112 to -0.004). Compared to the mMSKCC (C-index, 0.612) risk models, Model A and B showed significantly better predictive ability (mean difference was -0.097, 95% CI -0.153 to -0.043, mean difference was -0.117, 95% CI -0.174, -0.066, respectively).

There were no significant increases of predictive ability in models with adding inflammatory factors to Heng risk model. On the other hand, the addition of inflammatory factors to mMSKCC risk model showed significant increases of predictive ability. Incorporating the neutrophil and AST into the mMSKCC risk model and NLR, alkaline phosphatase and DRR into the mMSKCC risk model showed that the C-index increased from 0.612 to 0.658 and 0.691, respectively.

## 4. Discussion

Development of an accurate prognostic model is important for a patient's risk-oriented treatment strategy in treatment-naive clinical settings. The current Heng and MSKCC models can potentially be improved by incorporating novel prognostic variables or can be replaced with new models with different variables [4, 5]. Our study evaluated the potential for novel prognostic factors to improve the predictive power of the current Heng and MSKCC risk models or to derive a new model entirely; to that end, we achieved a significant improvement in the predictive accuracy of OS. Notably, our new model plus the addition of new prognostic factors to current models reflects the importance of inflammatory factors; moreover, they were based on an Asian population, whereas the original MSKCC and Heng models are mainly based on Western populations and do not incorporate inflammation/immune-related factors. Our study thus offers wider applicability with more precise prognostication of patients of different ethnicities.

A number of factors analyzed in our study have already been shown to significantly predict PFS and OS [13]. The most interesting finding in our study was that the NLR, DRR (or AST) and nephrectomy were significant prognostic factors for both PFS and OS. Our results also reflect the limitation of the current MSKCC and Heng risk models, in which PFS and OS are not always correlated with each other [19].

The prognostic significances of nephrectomy, NLR, and DRR were previously described [14, 20, 21]; however, no study has previously demonstrated their collective implications for PFS and OS. The NLR and nephrectomy are the most common prognostic factors in mRCC; nephrectomy was incorporated into the recently revised Heng risk model [22]. NLR was also proposed as a replacement for the neutrophil count, and our findings demonstrated its superiority.

A partial rationale for our study was that RCC has been closely linked to immune responses in systemic inflammation [9]; moreover, cancerous tissues show a greater rate of aerobic glycolysis than normal tissue (the Warburg effect) [23]. Neutrophils are the major inflammatory component of tumors; circulating neutrophils produce cytokines that stimulate cancer progression [24], while tumor-associated neutrophils and their bone marrow precursors (peripheral neutrophils and myeloid positive suppressor cells) suppress immune T cells [25]. The association of increased neutrophil counts with poor RCC prognosis [1] resulted in elevated neutrophils being considered an independent predictor of poor prognosis in the Heng risk model of clear-cell mRCC [5] and non-mRCC [16] during treatment [21, 26]. The switch from neutrophil count to NLR was based on the idea that the latter is a potential indicator of host immune and neutrophil-dependent tumorigenesis, as well as inflammation induced by T cell function [20]. Patients with an increased NLR exhibit relative lymphocytopenia, which can lead to worse prognosis and an increased potential for tumor progression.

The baseline NLR and its changes during targeted therapy administration may predict outcomes, as early NLR decrease was associated with favorable PFS and OS whereas its increase was associated with unfavorable outcomes [15]. This can assist clinicians in determining whether to maintain treatment with the same therapeutic agent or switch to another (e.g., in patients whose tumors slightly grew on imaging [stable disease status] but with a drop in the NLR). Moreover, as tyrosine kinase inhibitors exert antiangiogenic and immunomodulatory effects such as neutrophil migration and T lymphocyte-dendritic cell cross-talk [27], the implications of NLR changes in mRCC patients receiving such therapies may have greater significance than in the RCC patient population as a whole. The NLR might also be useful when administering immunotherapeutic regimens [3].

We showed that the pretreatment DRR (or AST) is an independent predictive biomarker for PFS and OS in patients with mRCC treated with targeted therapy. Pathological processes that can lead to a higher proliferative state, tissue damage, and high tumor cell turnover tend to increase AST but not the liver-specific ALT (at least not to the same extent), making the AST/ALT ratio an attractive potential biomarker [28]. AST is expressed in different subcomponents of breast cancer, pancreatic cancer, lung cancer, and cholangiocarcinoma cells [28]. The DRR has already been suggested as an independent prognostic biomarker, including metastasis-free survival and OS after curative nephrectomy [14] for non-mRCC patients and those with mRCC who underwent cytoreductive nephrectomy [29].

Previous studies suggested explanations for the DRR's ability to predict survival in patients with RCC [14, 23]. The AST and ALT levels might be involved in glycolysis in clear-cell RCC. Moreover, von Hippel-Lindau loss, a key trigger of clear-cell RCC, elevates hypoxia-induced factor levels, which is linked to markedly increased glycolysis [30]. Moreover, AST is a critical component of the malate-aspartate shuttle pathway of glycolysis [30].

This study had several limitations, including its retrospective design, single center restrictions, and disproportionally small risk groups. The cut-off levels of NLR and DRR are arbitrary, so there were additional limitations to use as continuous variables. However their prognostic values ought to be sustained in further studies, as no standard guidelines currently exist for NLR cut-off values. Additionally, other inflammatory factors such as C-reactive proteins, interleukin-6, and gamma-glutamyltransferase should be considered in future studies. Finally, our new model does not include biomarkers or genomic information; more specific targets ought to be selected.

## 5. Conclusion

In overall survival, predictive ability was increased when NLR and DRR markers were added to established Heng or mMSKCC risk models in patients with mRCC treated with first-line targeted therapy. We observed significantly improved predictive ability over the established models, suggesting that our inflammatory factors ought to be incorporated into the Heng and MSKCC risk models.

## Figures and Tables

**Table 1:** Comparison of baseline clinicopathological demographics among treatment groups (N=158).

Variables	N (%) or mean±sd or median (min-max)
Age (miss=1, years)	58.62±10.64
Gender, Male/Female	124 (78.5)/34 (21.5)
Metastatic type, Synchronous/Metachronous	97 (61.4)/61 (38.6)
Body mass index (miss=13)	23.70±3.27
KPS (miss=19), KPS >80	127 (91.4)
KPS ≤80	12 (8.6)
Nephrectomy	89 (56.3)
ECOG baseline (miss=1) 0/1+2+3/unknown	75 (47.8)/64 (40.8)/18 (11.5)
Underlying disease, Diabetes (miss=1)	37 (23.6)
Hypertension (miss=1)	73 (46.5)
Cerebrovascular disease	6 (3.8)
Cardiac disease	4 (2.5)
Duration from the first-line treatment (months)	4.8 (1.0-70.4)
Disease free interval (months)	2.0 (0.0-240.0)
Disease free interval≤1 year	106 (67.1)
MSKCC new (miss=52) favorable/intermediate/poor	13 (12.3)/75 (70.8)/18 (17)
Heng new (miss=26) favorable/intermediate/poor	14 (10.6)/94 (71.2)/24 (18.2)
Metastatic Organ, Lung metastasis	113 (71.5)
Liver metastasis (miss=1)	33 (21)
Lymph node metastasis	69 (43.7)
Bone metastasis (miss=1)	54 (34.4)
Brain metastasis (miss=5)	18 (11.8)
Number of metastatic organs (miss=6)	2.20±0.96
Baseline laboratory parameters	
Leukocyte (miss=4) ≥10	29 (18.8)
Hemoglobin (miss=4) M<13, F<11.5	90 (58.4)
Platelet (miss=4) ≥400K	19 (12.3)
Neutrophil (miss=6) <7500/	124 (81.6)
Neutrophil lymphocyte ratio	2.68 (0.77-39.2)
Lymphocyte (miss=4) ≥1500	94 (61)
LDH (miss=41) ≥300	13 (11.1)
Corrected Calcium (miss=9) ≥10	11 (7.4)
Albumin (miss=10) <3.5	23 (15.5)
Alkaline phosphatase (miss=14) ≥104	50 (34.7)
AST (miss=10) ≥40	12 (8.1)
ALT (miss=10)≥40)	18 (12.2)
De Retis ratio	1.38±0.92
Creatinine (miss=7) ≥0.9	134 (88.7)
Targeted agents TKI (miss=1), sunitinib, sorafenib, pazopanib	105 (66.9)/21 (13.4)/31 (19.8)
First line treatment result continue/PD/AE/unknown	9 (5.7)/129 (81.7)/11 (7)/9 (5.7)
Survival (%)	17.70%
Progression (%)	98.10%

KPS, Karnofsky performance status score; ECOG, Eastern Cooperative Oncology Group performance status; MSKCC, Memorial Sloan Kettering Cancer Center; LDH, lactate dehydrogenase; AST, aspartate transaminase; ALT, alanine transaminase.

**Table 2:** Univariate and multivariate analyses of the new prognostic factors for progression-free survival.

Variables	Univariable			Multivariable model 1		Multivariable model 2	
	N (event)	HR (95% CI)	p-value	HR (95% CI)	p-value	HR (95% CI)	p-value
Age ≥55 years	101 (76)	0.79 (0.56-1.13)	0.204				
Female gender	34 (28)	1.07 (0.69-1.65)	0.770				
Metachronous type	61 (50)	0.64 (0.44-0.92)	0.015				
Nephrectomy	89 (72)	0.48 (0.33-0.70)	<.001	0.48 (0.32-0.71)	<.001	0.48 (0.33-0.71)	<.001
Body mass index	145 (121)	0.98 (0.92-1.04)	0.500				
KPS≤80	12 (11)	1.10 (0.59-2.08)	0.760				
DFI≤1year	106 (87)	1.76 (1.20-2.58)	0.004				
mMSKCC, favorable	48 (42)	1	(0.287)				
intermediate	74 (59)	1.38 (0.92-2.06)	0.121				
poor	12 (10)	1.34 (0.66-2.71)	0.415				
Heng, favorable	14 (12)	1	(0.021)				
intermediate	94 (77)	2.00 (1.08-3.73)	0.029				
poor	24 (20)	2.84 (1.36-5.92)	0.006				
Lung metastasis	113 (93)	0.79 (0.53-1.16)	0.228				
Liver metastasis	33 (26)	1.23 (0.79-1.90)	0.366				
Bone metastasis	54 (47)	0.92 (0.64-1.32)	0.652				
Brain metastasis	18 (16)	1.30 (0.77-2.22)	0.329				
Hb, M<13, F<11.5	90 (70)	1.30 (0.91-1.86)	0.148				
Platelet ≥400K	19 (15)	1.95 (1.12-3.39)	0.018				
Neutrophil ≥7500	28 (21)	1.60 (0.99-2.57)	0.054				
Lymphocyte≥1500	60 (47)	1.37 (0.95-1.98)	0.097				
NLR	152 (125)	1.03 (1.00-1.06)	0.026			1.04 (1.00-1.07)	0.029
LDH ≥300	13 (12)	1.72 (0.93-3.19)	0.084				
Corrected Calcium ≥10	11 (9)	0.93 (0.46-1.85)	0.832				
Albumin <3.5	23 (16)	1.78 (1.04-3.07)	0.037				
Alkaline phosphatase ≥104	50 (42)	1.40 (0.96-2.07)	0.085				
AST≥40	12 (11)	2.56 (1.36-4.84)	0.004	1.96 (1.03-3.76)	0.042		
ALT≥40	18 (15)	1.26 (0.73-2.18)	0.399				
De Retis ratio	148 (122)	1.19 (0.97-1.45)	0.096				

KPS, Karnofsky performance status score; DFI, disease-free interval; Hb, hemoglobin; NLR, neutrophil-to-lymphocyte ratio; LDH, lactate dehydrogenase; AST, aspartate transaminase; ALT, alanine transaminase; HR, hazard ratio; CI, confidence interval.

Multivariable model 1 (uni p-value ≤0.15 without LDH) used with metachronous type, nephrectomy, DFI<1, Hb, platelet, neutrophil, lymphocyte, albumin, Alkaline phosphatase, AST.

Multivariable model 2 (uni p-value ≤0.15 without LDH) used with metachronous type, nephrectomy, DFI<1, Hb, platelet, albumin, Alkaline phosphatase, NLR, de retis ratio.

**Table 3:** Univariate and multivariate analyses of overall survival using the new prognostic factors.

Variables	Univariable			Multivariable 1		Multivariable 2	
	N (event)	HR (95% CI)	p-value	HR (95% CI)	p-value	HR (95% CI)	p-value
Age ≥55 years	101 (81)	0.92 (0.64-1.32)	0.635				
Female gender	34 (33)	1.38 (0.92-2.06)	0.117				
Metachronous type	61 (48)	0.48 (0.33-0.69)	<.001				
Nephrectomy	89 (70)	0.34 (0.23-0.50)	<.001	0.37 (0.24-0.55)	<.001	0.38 (0.25-0.56)	<.001
Body mass index	145 (118)	0.96 (0.90-1.02)	0.159				
KPS≤80	12 (10)	1.02 (0.53-1.96)	0.955				
DFI≤1year	106 (88)	2.04 (1.40-2.99)	<.001				
mMSKCC, favorable	48 (33)	1	(0.003)				
intermediate	74 (64)	1.77 (1.16-2.70)	0.009				
poor	12 (12)	2.83 (1.45-5.54)	0.002				
Heng, favorable	14 (7)	1	<.001				
intermediate	94 (79)	3.11 (1.42-6.78)	0.004				
poor	24 (21)	6.46 (2.70-15.46)	<.001				
Lung metastasis	113 (91)	0.76 (0.52-1.11)	0.152				
Liver metastasis	33 (32)	1.92 (1.28-2.89)	0.002	1.88 (1.21-2.94)	0.005		
Bone metastasis	54 (48)	1.12 (0.78-1.61)	0.533				
Brain metastasis	18 (13)	0.98 (0.55-1.75)	0.955				
Hb, M<13, F<11.5	90 (81)	2.04 (1.41-2.94)	<.001	1.83 (1.23-2.71)	0.003	1.71 (1.16-2.51)	0.007
Platelet ≥400	19 (16)	2.51 (1.45-4.34)	0.001				
Neutrophil ≥7500	28 (24)	2.17 (1.37-3.42)	0.001	2.58 (1.55-4.30)	<.001		
Lymphocyte≥1500	60 (53)	1.71 (1.19-2.45)	0.003				
NLR	152 (125)	1.06 (1.03-1.09)	<.001			1.07 (1.04-1.11)	<.001
LDH ≥300	13 (11)	1.37 (0.72-2.58)	0.338				
Corrected Calcium ≥10	11 (11)	1.73 (0.92-3.24)	0.087				
Albumin <3.5	23 (21)	3.77 (2.28-6.22)	<.001				
Alkaline phosphatase≥104	50 (43)	1.83 (1.23-2.70)	0.003	1.63 (1.08-2.45)	0.019	1.73 (1.16-2.58)	0.008
AST≥40	12 (12)	3.60 (1.95-6.65)	<.001				
ALT≥40	101 (81)	0.92 (0.64-1.32)	0.635				
De Retis ratio	34 (33)	1.38 (0.92-2.06)	0.117			1.34 (1.09-1.64)	0.006

KPS, Karnofsky performance score; DFI, disease-free interval; Hb, hemoglobin; NLR, neutrophil-to-lymphocyte ratio; LDH, lactate dehydrogenase; AST, aspartate transaminase; ALT, alanine transaminase; HR, hazard ratio; CI, confidence interval.

Multivariable 1 (uni p-value ≤0.15 without LDH) used with gender, metachronous type, nephrectomy, DFI<1, liver mets, Hb, platelet, neutrophil, Lymphocyte, corrected ca, Alkaline phosphatase, AST.

Multivariable 2 (uni p-value ≤0.15 without LDH) used with gender, metachronous type, nephrectomy, DFI<1, liver mets, Hb, platelet, corrected ca, Alkaline phosphatase, NLR, de retis ratio.

**Table 4:** Comparison of new risk models for progression-free survival using the Heng and MSKCC risk models with 2000 bootstraps.

Model	Harrell's C index	mean(difference), 95% CI (2.5%, 97.5% of difference)
Model A	0.594	Model B vs A: 0.017 ( -0.021, 0.057)
Model B	0.610	

Heng risk model	0.614	Heng vs Model A: 0.034 ( -0.030, 0.103)
		Heng vs Model B: -0.009 ( -0.081, 0.058)
MSKCC risk model	0.569	mMSKCC vs Model A: -0.025 (-0.106, 0.054)
		mMSKCC vs Model B: -0.042 (-0.127, 0.036)

Heng risk model + DRR	0.639	Heng vs (Heng+DRR): -0.025 ( -0.082, 0.013)

Model C = mMSKCC risk model + AST	0.569	mMSKCC vs Model C -0.013 (-0.052, 0.015)
Model D = mMSKCC risk model + NLR + DRR	0.602	mMSKCC vs Model D: -0.046 (-0.117, 0.002)
		Model C vs Model D: -0.033 (-0.102, 0.020)

MSKCC, Memorial Sloan Kettering Cancer Center; AST, aspartate transaminase; NLR, neutrophil-to-lymphocyte ratio; CI, confidence interval.

Model A = Nephrectomy, AST.

Model B = Nephrectomy, NLR.

**Table 5:** Comparison of new risk models for overall survival using the Heng and MSKCC risk models with 2000 bootstraps.

Model	Harrell's C index	mean(difference), 95% CI (2.5%, 97.5% of difference)
Model A	0.708	Model B vs A: 0.02 (-0.011, 0.058)
Model B	0.727	

Heng risk model	0.661	Heng vs Model A: -0.035 (-0.088, 0.008) Heng vs Model B: -0.055 (-0.112, -0.004)
mMSKCC risk model	0.612	mMSKCC vs Model A: -0.097 (-0.153, -0.043) mMSKCC vs Model B: -0.117 (-0.174, -0.066)

Model C = Heng risk model + AST	0.676	Heng vs (Heng + SGOT): -0.011 (-0.031, 0.004)
Model D = Heng risk model + Alkaline phosphatase + DRR	0.697	Heng vs (Heng + De Ritis ratio): -0.035 (-0.083, 0)
		(Heng + SGOT) vs (Heng + De Ritis ratio): -0.024 (-0.07, 0.011)

Model E = mMSKCC risk model + Neutrophil + AST	0.658	mMSKCC vs Model E: -0.049 (-0.098, -0.013)
Model F = mMSKCC risk model + NLR + Alkaline phosphatase +DRR	0.691	mMSKCC vs Model F: -0.084 (-0.149, -0.034) Model E vs Model F: -0.034 (-0.092, 0.014)

MSKCC, Memorial Sloan Kettering Cancer Center; AST, aspartate transaminase; NLR, neutrophil-to-lymphocyte ratio; CI, confidence interval.

Model A = Nephrectomy, Liver mets, Hb, Neutrophil, Alkaline phosphatase.

Model B = Nephrectomy, Hb, NLR, Alkaline phosphatase, DRR.

## Data Availability

The datasets used and/or analyzed during the current study are available from the corresponding author (Jinsoo Chung, cjs5225@ncc.re.kr) on reasonable request. The IRB and ethical committee of the National Cancer Center (in Korea) will review the requests because of the patients' information. After the approval of the committee with confirmation of the reasonable requests, the dataset will be freely available. The other contact e-mail besides the corresponding author's e-mail is irb@ncc.re.kr.
